# A new portable monitor for measuring odorous compounds in oral, exhaled and nasal air

**DOI:** 10.1186/1472-6831-11-15

**Published:** 2011-04-20

**Authors:** Naofumi Tamaki, Kenta Kasuyama, Mitsue Esaki, Takara Toshikawa, Shun-Ichi Honda, Daisuke Ekuni, Takaaki Tomofuji, Manabu Morita

**Affiliations:** 1 Department of Preventive Dentistry, Okayama University Graduate School of Medicine, Dentistry and Pharmaceutical Sciences, Okayama, Japan; 2 Department of Preventive Dentistry, Division of Oral Health Science, Hokkaido University Graduate School of Dental Medicine, Sapporo, Japan; 3 TAIYO Instrument INC, Osaka, Japan; 4 Honda Dental Clinic, Higashi-Osaka, Japan

## Abstract

**Background:**

The B/B Checker^®^, a new portable device for detecting odorous compounds in oral, exhaled, and nasal air, is now available. As a single unit, this device is capable of detecting several kinds of gases mixed with volatile sulfur compounds (VSC) in addition to other odorous gasses. The purpose of the present study was to evaluate the effectiveness of the B/B Checker^® ^for detecting the malodor level of oral, exhaled, and nasal air.

**Methods:**

A total of 30 healthy, non-smoking volunteers (16 males and 14 females) participated in this study. The malodor levels in oral, exhaled, and nasal air were measured using the B/B Checker^® ^and by organoleptic test (OT) scores. The VSCs in each air were also measured by gas chromatography (GC). Associations among B/B Checker^® ^measurements, OT scores and VSC levels were analyzed using Spearman correlation coefficients. In order to determine the appropriate B/B Checker^® ^level for screening subjects with malodor, sensitivity and specificity were calculated using OT scores as an identifier for diagnosing oral malodor.

**Results:**

In oral and nasal air, the total VSC levels measured by GC significantly correlated to that measured by the B/B Checker^®^. Significant correlation was observed between the results of OT scores and the B/B Checker^® ^measurements in oral (r = 0.892, p < 0.001), exhaled (r = 0.748, p < 0.001) and nasal air (r = 0.534, p < 0.001). The correlation between the OT scores and VSC levels was significant only for oral air (r = 0.790, p < 0.001) and nasal air (r = 0.431, p = 0.002); not for exhaled air (r = 0.310, p = 0.096). When the screening level of the B/B Checker^® ^was set to 50.0 for oral air, the sensitivity and specificity were 1.00 and 0.90, respectively. On the other hand, the screening level of the B/B Checker^® ^was set to 60.0 for exhaled air, the sensitivity and specificity were 0.82 and 1.00, respectively.

**Conclusion:**

The B/B Checker^® ^is useful for objective evaluation of malodor in oral, exhaled and nasal air and for screening subjects with halitosis.

**Trial registration:**

ClinicalTrials.gov: NCT01139073

## Background

Halitosis affects a large proportion of the global population and may be the cause of a significant social or psychological handicap [1]. The number of subjects who visit dental clinics complaining of halitosis has been increasing, and it is estimated that more than 50% of the population in North America suffer from halitosis [2,3]. An epidemiological survey of the general population of Japan showed that 24% of the individuals examined complained about bad breath [4].

Much emphasis has been reported in the literature regarding unpleasant odors in the oral cavity. Volatile sulfur compounds (VSCs) generated by oral bacteria are the major causes of halitosis and are major components of intra-oral odors [5,6]. On the other hand, nasal passages predominate among extra-oral etiologies of bad breath. Nasal malodor is indicative of a nasal infection, such as sinusitis, tonsillitis, and rhinitis [7]. It has also been reported that patients with cleft lip and/or palate have a greater tendency to present nasal malodor compared to those without cleft lip and/or palate [8]. Moreover, odorous gases emanating form the pharyngeal or bronchial region contaminate exhaled air from the lungs [9]. Therefore, in order to manage patients with halitosis, an accurate and objective instrument for measuring malodor level in oral, exhaled, and nasal air is required. To date, however, only a few effective instruments like the halimeter exist that are capable of measuring malodor level originating from oral, exhaled and nasal air [7].

Currently, gas chromatography (GC) for determining the concentrations of VSCs is considered to be the most reliable method for measuring oral malodor [10]. Unfortunately, since GC is not suitable in daily clinical practice because of its complexity, simpler devices for detecting VSCs have been developed and are widely used [11-13]. Actual biological gas, however, contains several odorous compounds other than VSCs, such as n-dodecanol, phenol, indole, and diamine [14,15]. Indeed, it is commonly observed that VSC levels do not always correspond to actual odor levels perceived by humans. Therefore, organoleptic measurement, the subjective measurement of halitosis, is considered to be the most reliable, due to the fact that the human sense of smell can simultaneously detect several kinds of compounds, including VSCs and other odorous gases [3,16].

Recently, the B/B Checker^®^, a new portable gas detector for measuring the malodor level from human oral, exhaled, and nasal air has been developed. As a single unit, this device is capable of detection of several kinds of gases, and also measuring of the malodor level in oral, exhaled, and nasal gases independently in a short period of time. The purpose of the present study was to examine the effectiveness of this new device in measuring malodor level in oral, exhaled and nasal air.

## Methods

### Subjects

A total of 30 periodontal healthy, non-smoking volunteers (16 males and 14 females, mean age: 43.9 ± 18.5 years) without medical disorders, and not undergoing any antibiotic or other antimicrobial therapy participated in the present study.

### Measurement of malodor compounds

The following three gases from different origins were measured to determine malodor level: oral gas, which stagnates in the oral cavity (mouth air); exhaled gas, which passes through the oral cavity from the lungs following expiration (exhaled air); and nasal gas, which is expelled through the nasal cavity when the mouth is closed (nasal air). To determine genuine malodor level, the subjects were advised to abstain from food or drink on the morning of the assessment day and to refrain from their usual oral hygiene practice the morning of the day of the measurements. Subjects were also instructed to refrain from eating strong smelling foods for at least 48 h, using strong perfumes for 24 h, and drinking alcohol for 12 h prior to the measurements. Actual measurements were conducted in the morning, between 11:00 and 12:00. Subjects kept their mouths closed for 3 min prior to the measurement of oral malodor [13]. Before measuring exhaled and nasal air, subjects were instructed to keep their mouths open and breathe through the mouth for 30 sec.

### B/B Checker^® ^measurement

**Figure 1:**
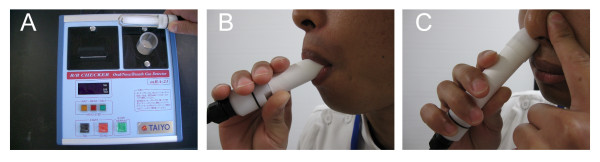
**A new portable malodor monitor, the B/B Checker^®^**. (A) The size of the B/B Checker is W 210 mm × D 230 mm × H 80 mm. The output value is indicated automatically and printed using a built-in printer. (B) Measurement of oral gas and exhaled gas. (C) Measurement of nasal gas.

A thin-coat tin dioxide semiconductor gas sensor, that is sensitive to reductive gases, has been developed by Taiyo Instrument Inc., Osaka, Japan, and comprises a sensor probe and a main body equipped with a printer (Figure 1A). The sensor will detect various gasses, such as VSCs, hydrogen, ethanol, acetone, butylate, and ammonia. The B/B Checker^® ^expresses the gas level (B/B Value) as the olfactory intensity of human according to the Weber-Fechner law. The gas level as the olfactory intensity was outputted as a single value (Range 0-100). The Weber-Fechner law states that the magnitude of a subjective sensation increases proportional to the logarithm of the stimulus intensity. In this case, I = k*logC, where I (B/B value) is the magnitudes of the perceived intensity of the stimuli (odor) and C is the magnitude of stimuli (concentration of odorous compounds).

The sensor probe covered with disposable adaptor was inserted directly into the mouth to prevent the loss of sample gas (Figure 1B). The cover prevents the direct contact of oral mucosa to sensor probe. Thus, there is little interference with gases in the oral cavity from surrounding gases during the measurement procedure and the sensor is capable of directly determining the gas levels without any interposition. Before oral air analysis, subjects were instructed to keep their mouths closed and to breathe through the nose for 180 sec. The sensor was inserted into the center of the oral cavity. Oral air was measured for 15 sec.

At the measurement of exhaled breath, the subjects were at first asked to expel oral air. The subjects breathed a deep inspiration, held their breath for 15 sec, and then tried to exhale breath from the lungs completely through the adapter over 15 sec. The B/B Checker^® ^is capable of measuring maximum odorous gas levels in the terminal lung gas.

The sensor probe was then inserted into the nose adapter to measure nasal air. The malodor level in air from the right and left nasal cavity was analyzed separately. To measure the malodor level from one nasal cavity, the subjects compressed the opposite nostril with a finger. (Figure 1C).

### Gas chromatography analysis

The GC analysis was conducted using a GC-14B gas chromatograph (Shimadzu Co., Kyoto, Japan), equipped with a flame photometric detector. After subjects kept their mouths closed for 3 min, a Teflon^® ^tube connected to a glass syringe was inserted into the center of the oral cavity through the lips and teeth, while the lips remained closed. When measuring the malodor level of exhaled air, the gas samples were first collected in a Gas Pack, and a Teflon^® ^tube was used for aspiration. Measurement of nasal air malodor was performed by inserting the Teflon^® ^tube connected to a glass syringe approximately 1 cm into each nostril [17]. Following aspiration of 10 ml of oral, exhaled and nasal air, a 5 ml sample of air was transferred to the Shimalite^® ^TPA column and gas chromatograph. The VSCs, hydrogen sulfide, methyl mercaptan, and dimethyl sulfide were determined by their characteristic retention time and were quantified via comparison of their peak area with that of dilutions of standards [18].

### Organoleptic test

For the organoleptic test (OT), subjects were asked to exhale the gas through their mouth or nose briefly with moderate force at a distance of approximately 10 cm from the examiner. The malodor level in mouth and nasal air was subsequently assessed. When measuring the malodor level of exhaled air, the gas collected in the Gas Pack for GC analysis was assessed. The organoleptic measurement was performed by two trained dentists (KK, ME). These examiners rated the malodor on a 0-5 scale according to the procedures outlined in a previous report [19,20]. Briefly, a score of 0 represented the absence of malodor, 1 barely noticeable malodor, 2 slight malodor, 3 moderate malodor, 4 strong malodor, and 5 extremely strong malodor. Examiners were blind to both the B/B Checker^® ^measurements and GC analyses. The inter-examiner correlation of oraganoleptic rating was 0.95 (p < 0.001).

### Statistical analysis

Data analysis was done with the Statistical Package for Social Science (SPSS version 19, SPSS Japan, Tokyo, Japan). Kruskal Wallis test was used to investigate the differences of mean values of measurements among the gasses from different origins. The association among the B/B Checker^® ^measurements, OT scores and GC analysis of VSC concentrations was analyzed using Spearman correlation coefficients. In order to determine the appropriate level of the B/B Checker^® ^for screening subjects with malodor, sensitivity and specificity was calculated using OT scores (≥ 3) as an identifier for diagnosing oral malodor. The sample size was calculated using statistical software (nQuery Advisor, Statistical Solutions, Sangus, MA, USA) based on the differences in malodor parameters (Oral gas and Exhaled gas) between high (≥ 3) and low OT scores in our preliminary study. A sample size of 9 per group was required for detection of a significant difference in exhaled gas (80% power; two-sided 5% significance level).

### Ethical approval and registration

The protocol was approved by the Institutional Review Board of the Ethics Committee of Okayama University Graduate School of Medicine, Dentistry and Pharmaceutical Sciences (No.425). All subjects provided signed informed consent before entry into the study. The trial is registered with ClinicalTrials.gov protocol registration system, ID NCT01139073.

## Results

The mean level ± standard deviation of each gas measured using different procedures are shown in Table 1. Since methyl mercaptan and dimethyl sulfide were not detected in most of subjects, the measurement using GC was expressed as hydrogen sulfide level and total concentration of VSCs. All measurement procedures determined that exhaled air was the most odorous, followed by oral air and nasal air. All odor levels, i.e., B/B Checker^® ^measurements, OT scores, and GC analyses for nasal air were significantly lower than those for oral and exhaled air. There was no significant difference of malodor level between oral air and exhaled air.

**Table 1:** Malodor measurements in oral, exhaled and nasal air.

			Gas Chromatography (ppb)	
			
	Organoleptic score	B/B Checker (B/B value)	Hydrogen sulfide	Total VSCs
Oral air (N = 30)	2.6 ± 1.1	55.5 ± 18.3	77.9 ± 43.3	86.9 ± 47.6

Exhaled air (N = 30)	2.8 ± 1.0	61.4 ± 16.1	99.8 ± 65.8	106.4 ± 63.6

Nasal air (N = 60^#^)	1.3 ± 0.9*	41.0 ± 18.1*	46.9 ± 24.6*	49.1 ± 24.5*

Mean ± Standard deviation

^# ^Right and left nasal air was measured individually

* Significantly lower than oral and exhaled air, p < 0.001

**Figure 2:**
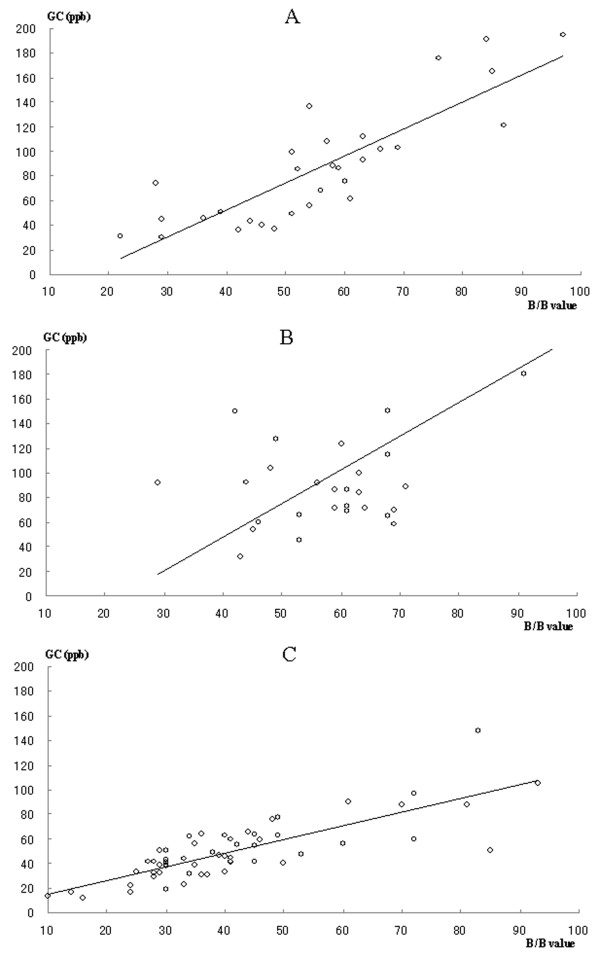
**Correlation between the results of B/B Checker measurements and total VSCs measured by GC**. (A) Oral gas; r = 0.843, p < 0.001 (N = 30), (B) Exhaled gas; r = 0.190, p = 0.314 (N = 30), (C) Nasal gas; r = 0.783, p < 0.001 (N = 60).

The correlation between the B/B Checker^® ^measurements and total concentration of VSCs is shown in Figure 2. In oral and nasal air, total concentration of VSCs measured by GC significantly correlated to the malodor level detected using the B/B Checker^®^. However, there was no significant relationship between the two measurements in exhaled air.

Since the organoleptic scoring method is still considered to be the gold standard for determining oral malodor, the correlation coefficients of OT scores with the B/B Checker^® ^and total concentration of VSCs measured by GC was calculated (Table 2). In all three types of air, a significant correlation was observed between the OT scores and B/B Checker^® ^measurements (oral air: 0.892, p < 0.001; exhaled air: 0.748, p < 0.001; and nasal air: 0.534, p < 0.001). On the other hand, the correlation between the OT scores and total concentration of VSCs was significant only for oral air (0.790, p < 0.001) or nasal air (0.431, p = 0.002); not for exhaled (0.310, p = 0.096).

**Table 2:** Correlation coefficients of organoleptic ratings with the B/B Checker measurements and total VSCs measured by GC.

	Correlation coefficients of organoleptic ratings with	
	
	B/B Checker	total VSCs by GC
Oral air	0.892***	0.790***

Exhaled air	0.748***	0.310

Nasal air	0.534***	0.431**

**: p < 0.01, ***: p < 0.001

As shown in Table 1 the value of malodor level was relatively low. In order to examine the usability of B/B Checker^® ^in halitosis patients, those with OT scores (≥ 3) were selected (n = 20). In oral air, a significant correlation was observed between concentration of hydrogen sulfide and B/B Checker^® ^measurements (0.790, p < 0.001).

**Figure 3:**
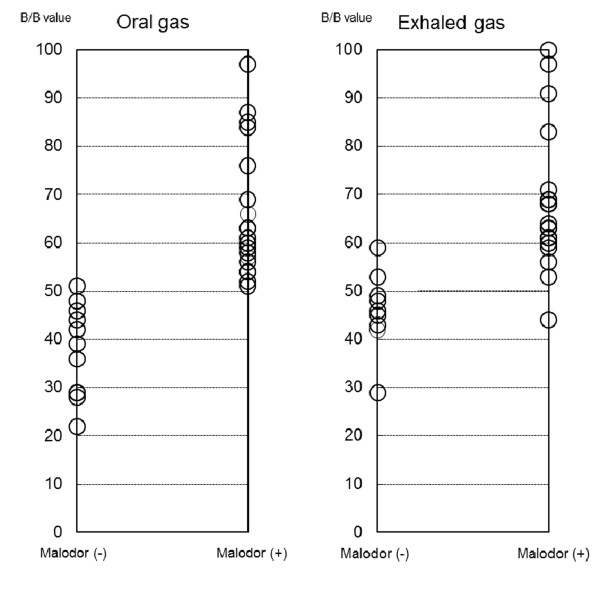
**Distribution of B/B Checker results according to the presence of noticeable organoleptic malodor**. Plots represent B/B value in each subject. The subjects with OT scores (≥ 3) was defined as those with clearly noticeable malodor.

The appropriate level of the B/B Checker^® ^for screening subjects with malodor was determined by calculating sensitivity and specificity. The subjects with OT scores (≥ 3) were defined as those with clearly noticeable malodor (Figure 3). When the screening level of the B/B Checker^® ^was set to 50 for oral air, the sensitivity and specificity was 1.00 and 0.90, respectively. On the other hand, when the screening level of the B/B Checker^® ^was set to 60 for exhaled air, the sensitivity and specificity was 0.82 and 1.00, respectively. Since there were few subjects with OT scores (≥ 3) in nasal air, this particular analysis was not performed.

## Discussion

The correlation coefficients of OT scores with the B/B Checker^® ^measurements were significant for the oral, exhaled, and nasal air. It is now commonly accepted that the organoleptic scoring method is a gold standard for the study of malodor. Moreover, the B/B Checker^® ^has relatively high sensitivity and specificity for screening subjects with noticeable malodor. Ueno et al. (2008) developed a new portable sulfide monitor, and reported that sensitivity is 0.8-0.9 and specificity is 0.6-0.8 [12]. The study by Ueno et al. employed 475 subjects, whereas only 30 participated in the present study. Although the high sensitivity (0.82-1.00) and specificity (0.90-1.00) of the B/B Checker^® ^must be re-evaluated in a larger population, it may be useful for objective evaluation of malodor in oral, exhaled and nasal air and for screening subjects with halitosis.

Significant association between OT scores and total concentration of VSCs by GC was observed only for oral air, and not for exhaled or nasal air. On the other hand, OT scores and B/B Checker^® ^measurements were significantly correlated for three kinds of air. In addition, there was no significant association between the total concentration of VSCs and B/B Checker^® ^measurements in exhaled air. While the reason remains unclear, the GC focuses only on VSCs; hydrogen sulfide, methyl mercaptan and dimethyl sulfide. Therefore, some of the major components of oral air are VSCs, substances that both the B/B Checker^® ^and GC are capable of detecting. It is likely that exhaled air, probably or nasal air, contain other major components that both the B/B Checker^® ^and human nose, and not GC, could detect. In fact, it has been hypothesized that indole, skatole, putrescine, and cadaverine, and some organic acids, like acetic and butyric acid, also contribute to malodor [15]. Recently, Van den Velde et al. (2009) reported that di- and trimethyl sulfide are associated with malodor [21]. Based on these findings, devices sensitive to several kinds of gasses are required for malodor study.

Tangeman and Winkel (2007) proposed to clearly differentiate the origin of halitosis, intra- and extra-orally. The most predominant sources of halitosis (80-90%) are present within the oral cavity and include bacterial reservoirs such as the dorsum of the tongue, saliva and periodontal pockets [22]. This corresponds to the malodor in oral air in the present study. However, less attention has been paid to extra-oral halitosis [22-24]. One of the origins of extra-oral halitosis is malodor in nasal air, which is considered to be related to serious systemic disease. Furthermore, we suspected that the air that passes through the oral cavity from the lung after expiring oral air that has stagnated in the oral cavity might be another origin of extra-oral halitosis. As a result, all three kinds of measurements, i.e., B/B Checker^® ^measurements, total concentration of VSCs, and OT scores, showed that the malodor level was greatest in the exhaled air, followed by oral air and nasal air. Since healthy subjects, without any medical disorders, were employed in the present study, the lowest scores in the nasal air were deemed reasonable. Moreover, the present study indicates that future studies should focus on exhaled air for halitosis management.

Many articles have discussed the generation of bad breath in the oral cavity. However, to date, few devices have been developed for effectively measuring malodor in nasal or exhaled air and few studies of nasal malodor and its causes have been published [17]. It has been reported that patients with cleft lip and/or palate have a greater tendency to present higher nasal VSC measurements, indicating the usefulness of VSC measurements for evaluation of quality of life (QOL) in these patients. The B/B Checker^® ^has a special adaptor for the nasal cavity, allowing the sensor to be placed directly into the nasal cavity without gas contamination. Accurate assessment and testing are important to determine which point is an actual problem. Therefore, the B/B Checker^® ^is applicable for QOL determination for cleft patients. The present study also examined the ability of conducting measurements of exhaled air in a clinical situation. Moreover, the B/B Checker^® ^showed adequate sensitivity for detecting malodor and a fair specificity for identifying non-malodor cases. The results of the present study suggest that the B/B Checker^® ^could be used as an adjunct instrument to a GC system and organoleptic methods currently in use at clinical situation for determining malodor in oral, exhaled and nasal air.

The malodor levels of exhaled air were higher than of oral air, however, no significant differences were observed in the levels of OT score, B/B value, hydrogen sulfide and VSCs. The sources of hydrogen sulfide may be multiple, including the bacterial flora [25], as well as endogenous sulfide-generating enzymes that exist in many cells and tissues of the human body [26]. Oral air was thought to be relatively low in subjects at the present study because periodontally healthy subjects were analyzed. If we have measured in malodor levels at periodontal patients, oral air would be higher than exhaled breath.

This study has some limitations. At first, the subjects who participated in the present study had relatively mild halitosis. The mean VSC levels ranged from 40 to 110 ppb of the total concentration of VSCs. It remains unclear whether the B/B Checker^® ^measurements correlate to GC or organoleptic ratings in subjects with severe halitosis. Since it is apparent that VSCs cause severe halitosis and that the B/B Checker^® ^is capable of detecting several gases, the B/B Checker^® ^might be less sensitive to severely malodorous air in 5 subjects complaining of oral malodor. Secondly, the present study is only a cross sectional study. Therefore, the effect of malodor management in clinic should be monitored using the B/B Checker^®^. Then, the usefulness of this device can be actually provided.

## Conclusion

The results from the present study show that the correlation coefficients of OT scores with measurements from the B/B Checker^® ^were significant for oral, exhaled and nasal air. In oral and nasal air, the total concentration of VSCs measured by GC significantly correlated to the malodor level determined using the B/B Checker^®^. The B/B Checker^® ^may be effective for objective evaluation of malodor in oral, exhaled and nasal air and for screening subjects with halitosis in the clinical setting. Further clinical studies are needed to investigate the capability of measuring the malodor of each type of air (i.e., oral, exhaled, nasal) in different sample groups with systemic disease or periodontal disease.

## Competing interests

One of the author (T. Toshikawa) is employees of Taiyo Instrument Inc., which has been involved in the B/B Checker^® ^development.

## Authors' contributions

NT has made substantial contribution to the study conception and design. KK, ME, TT^1^, DE, and SH implemented this study and participated in the acquisition, analysis and interpretation of data. TT^3 ^gave technical advices using the B/B Checker^®^. MM has been intimately involved in drafting and editing the manuscript. All authors read and approved the final manuscript.

## Pre-publication history

The pre-publication history for this paper can be accessed here:

http://www.biomedcentral.com/1472-6831/11/15/prepub
